# Seroprevalence of SARS-CoV-2 antibody among urban Iranian population: findings from the second large population-based cross-sectional study

**DOI:** 10.1186/s12889-022-13464-7

**Published:** 2022-05-23

**Authors:** Mohammad Zamani, Hossein Poustchi, Zahra Mohammadi, Sahar Dalvand, Maryam Sharafkhah, Seyed Abbas Motevalian, Saeid Eslami, Amir Emami, Mohammad Hossein Somi, Jamshid Yazdani-Charati, Nader Saki, Manoochehr Karami, Farid Najafi, Iraj Mohebbi, Nasrollah Veisi, Ahmad Hormati, Farhad Pourfarzi, Reza Ghadimi, Alireza Ansari-Moghaddam, Hamid Sharifi, Gholamreza Roshandel, Fariborz Mansour-Ghanaei, Farahnaz Joukar, Amaneh Shayanrad, Sareh Eghtesad, Ahmadreza Niavarani, Alireza Delavari, Soudeh Kaveh, Akbar Feizesani, Melineh Markarian, Fatemeh Shafighian, Alireza Sadjadi, Maryam Darvishian, Reza Malekzadeh

**Affiliations:** 1 Digestive Diseases Research Center, Digestive Diseases Research Institute, Shariati Hospital, Tehran University of Medical Sciences, Tehran, 14117–13135 Iran; 2 Liver and Pancreatobiliary Diseases Research Center, Digestive Diseases Research Institute, Shariati Hospital, Tehran University of Medical Sciences, Tehran, Iran; 3 Research Center for Addiction and Risky Behaviors (ReCARB), Psychosocial Health Research Institute, Iran University of Medical Sciences, Tehran, Iran; 4 Department of Epidemiology, School of Public Health, Iran University of Medical Sciences, Tehran, Iran; 5 Pharmaceutical Research Center, Pharmaceutical Technology Institute, Mashhad University of Medical Sciences, Mashhad, Iran; 6 Microbiology Department, Burn & Wound Healing Research Center, Shiraz University of Medical Sciences, Shiraz, Iran; 7 Liver and Gastrointestinal Diseases Research Center, Tabriz University of Medical Sciences, Tabriz, Iran; 8 Department of Biostatistics, Health Sciences Research Center, Addiction Institute, Faculty of Health, Mazandaran University of Medical Sciences, Sari, Iran; 9 Hearing Research Center, Ahvaz Jundishapur University of Medical Sciences, Ahvaz, Iran; 10 Department of Epidemiology, School of Public Health, Hamadan University of Medical Sciences, Hamadan, Iran; 11 Department of Epidemiology, School of Public Health & Safety, Shahid Beheshti University of Medical Sciences, Tehran, Iran; 12 Research Center for Environmental Determinants of Health, School of Public Health, Kermanshah UMS, Kermanshah, Iran; 13 Social Determinants of Health Center, Urmia University of Medical Sciences, Urmia, Iran; 14 Kurdistan University of Medical Sciences, Sanandaj, Iran; 15 Gastroenterology and Hepatology Disease Research Center, Qom University of Medical Science, Qom, Iran; 16 Department of Internal Medicine, School of Medicine, Gastrointestinal and Liver Diseases Research Center, Iran University of Medical Sciences, Tehran, Iran; 17 Digestive Disease Research Center, Ardabil University of Medical Sciences, Ardabil, Iran; 18 Social Determinants of Health Research Center, Health Research Institute, Babol University of Medical Sciences, Babol, Iran; 19 Health Promotion Research Center, Zahedan University of Medical Sciences, Zahedan, Iran; 20 HIV/STI Surveillance Research Center and WHO Collaborating Center for HIV Surveillance, Institute for Futures Studies in Health, Kerman University of Medical Sciences, Kerman, Iran; 21 Golestan Research Center of Gastroenterology and Hepatology, Golestan University of Medical Sciences, Gorgan, Iran; 22 Division of Gastroenterology & Hepatology, Gastrointestinal & Liver Diseases Research Center, Guilan University of Medical Sciences, Rasht, Iran; 23 Gastrointestinal and Liver Diseases Research Center, Guilan University of Medical Sciences, Rasht, Iran; 24 Digestive Oncology Research Center, Digestive Diseases Research Institute, Shariati Hospital, Tehran University of Medical Sciences, Tehran, Iran; 25 Cancer Control Research, BC Cancer Research Centre, Vancouver, BC Canada

**Keywords:** COVID-19, SARS-CoV-2, Seroprevalence, General population, Infection

## Abstract

**Background:**

The first large serosurvey in Iran found a SARS-CoV-2 antibody seroprevalence of 17.1% among the general population in the first wave of the epidemic by April, 2020. The purpose of the current study was to assess the seroprevalence of COVID-19 infection among Iranian general population after the third wave of the disease.

**Methods:**

This population-based cross-sectional study was conducted on 7411 individuals aged ≥10 years old in 16 cities across 15 provinces in Iran between January and March, 2021. We randomly sampled individuals registered in the Iranian electronic health record system based on their national identification numbers and invited them by telephone to a healthcare center for data collection. Presence of SARS-CoV-2-specific IgG and IgM antibodies was assessed using the SARS-CoV-2 ELISA kits. The participants were also asked about their recent COVID-19-related symptoms, including cough, fever, chills, sore throat, headache, dyspnea, diarrhea, anosmia, conjunctivitis, weakness, myalgia, arthralgia, altered level of consciousness, and chest pain. The seroprevalence was estimated after adjustment for population weighting and test performance.

**Results:**

The overall population-weighted seroprevalence adjusted for test performance was 34.2% (95% CI 31.0-37.3), with an estimated 7,667,874 (95% CI 6,950,412-8,362,915) infected individuals from the 16 cities. The seroprevalence varied between the cities, from the highest estimate in Tabriz (39.2% [95% CI 33.0-45.5]) to the lowest estimate in Kerman (16.0% [95% CI 10.7-21.4]). In the 16 cities studied, 50.9% of the seropositive individuals did not report a history of symptoms suggestive of COVID-19, implying an estimation of 3,902,948 (95% CI 3,537,760-4,256,724) asymptomatic infected individuals.

**Conclusions:**

Nearly one in three individuals were exposed to SARS-CoV-2 in the studied cities by March 2021. The seroprevalence increased about two-fold between April, 2020, and March, 2021.

**Supplementary Information:**

The online version contains supplementary material available at 10.1186/s12889-022-13464-7.

## Introduction

Since the beginning of the coronavirus disease 2019 (COVID-19) pandemic caused by the novel severe acute respiratory syndrome coronavirus 2 (SARS-CoV-2), more than 517 million COVID-19 cases and more than 6.2 million deaths have been reported around the world [1]. In the meantime, Iran was one of the first countries that had been affected by the virus outbreak. As of May 8, 2022, more than 7.2 million confirmed cases and more than 141 thousand deaths have been reported from the country [1]. However, the true number of infected cases is underestimated due to different factors, such as asymptomatic infection, variable management of mild cases, etc. Therefore, in addition to the case-based surveillance, conducting population-based seroepidemiological studies in a region is useful to measure the burden of COVID-19 infection and its fatality rate (by dividing the cumulative number of SARS-CoV-2 deaths by the number of individuals estimated to be infected), as well as the magnitude of the disease transmission over time [2].

In the first large population-based serosurvey in Iran, a seroprevalence rate of 17.1% was reported in the general population by the end of April, 2020 (first wave), with considerable variations in SARS-CoV-2 prevalence between the cities [3]. During the next months, the government has tried to limit the viral spread by regional lockdowns and social distancing policies [4]; however, Iran experienced the second (from mid-May to mid-August, 2020) and third (from early October 2020 to early January 2021) waves of the disease [1].

Monitoring the trend of seroprevalence of SARS-CoV-2 infection is necessary to reflect the latest status of the disease and to assess whether the social distancing policies were efficient in containing the SARS-CoV-2 spread [5]. In this study, we aimed to perform the second population-based cross-sectional study to investigate the seroprevalence rate of SARS-CoV-2 infection after the third wave of the epidemic in Iran, as well as to measure the changes in the seroprevalence across cities.

## Materials and methods

### Study design and participants

**Fig. 1:**
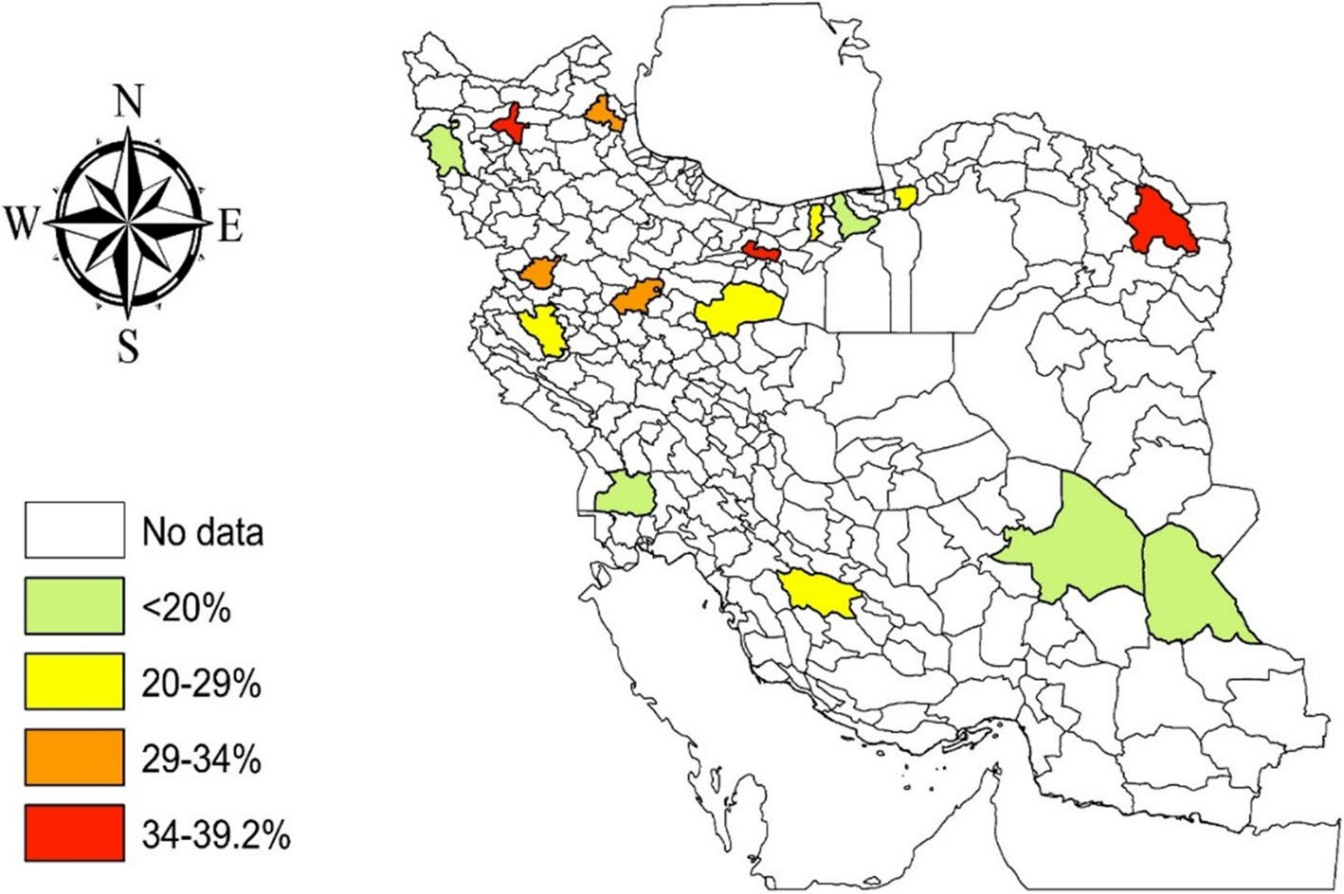
Seroprevalence (adjusted for test performance I) of severe acute respiratory syndrome coronavirus 2 in 16 cities included in the study. This map was created using ArcGIS software by Esri (http://www.esri.com)

This population-based cross-sectional study was conducted in 16 cities across 15 provinces in Iran, including Ardabil, Babol, Gorgan, Sari, Tabriz, and Urmia in the northern provinces, Hamedan, Kermanshah, Mashhad, Qom, Tehran, and Sanandaj in the central provinces, and Ahvaz, Kerman, Shiraz, and Zahedan in the southern provinces (Fig. 1). The detailed sampling method was described in the first study phase [3]. In brief, we randomly sampled the general population registered in the Iranian electronic health record system (SIB) based on their national identification numbers and invited them by telephone to refer to a healthcare center for data collection. SIB network belongs to a prospective population-based cohort study in which the demographic information and administrative health data for > 88% of Iranians (about 72 million people) are registered [6]. We included individuals who were aged ≥10 years old, and excluded those who were inaccessible or unwilling to participate in the study. Contrary to our previous serosurvey, we did not enroll high-risk individuals in the present study, that is, we did not include high-risk occupational groups (such as healthcare workers, etc.). We considered provincial capitals as clusters due to the heterogeneous pattern of COVID-19 dispersion across the provinces of Iran, as well as the factors such as population density, the high correlation of humidity in each province with COVID-19 prevalence, and intra-city and intra-provincial movements, which could affect the COVID-19 prevalence [7, 8].

### Sample size calculation

The sample size was calculated based on the estimated COVID-19 prevalence of 14.2% [9], a relative estimation error of 10%, considering a 5% precision, a non-response rate of 10%, and a design effect (Deff) of 1.75 to adjust for the nature of sampling by the following form:

Deff = 1 + d(n-1); where the intraclass correlation coefficient (d) was 0.05 and cluster (*n* = 16) was the total number of cities. The total sample size for this study by mentioned information was 9010 individuals. Sample size formulation was:

$$\mathrm{n}=\frac{\left({\mathrm{z}}_{1-\upalpha \left/ 2\right.}^2\right)\ast \mathrm{p}\ast \left(1-\mathrm{p}\right)}{{\mathrm{d}}^2}$$

### Procedures

After referring to a collaborating center, the participants were interviewed by trained research staff to complete questionnaires containing demographic details, past medical history, COVID-19-related symptoms, and COVID-19-related exposures. After collecting the required information, a 6 ml sample of venous blood was collected from each participant by a skilled laboratory technician into an EDTA-coated microtainer labeled with a unique participant identity number. Centrifuged plasma samples were then transported to a central laboratory on the dry ice (minus 20 degrees centigrade). Serum samples were assessed for the presence of SARS-CoV-2 nucleocapsid protein IgG and IgM antibodies, using Iran’s Food and Drug Administration-approved SARS-CoV-2 ELISA kits (Pishtaz Teb, Tehran, Iran) as per the manufacturer’s protocol [10]. The kits were designed based on indirect method in which SARS-CoV-2 specific nucleocapsid were coated in the 96-well plates. The recombinant SARS-CoV-2 nucleocapsid protein expressed in Baculovirus-insect cells consists of 1-419 amino acids and predicts a molecular mass of 47.08 kDa. The information on the sample collection and ELISA kits has been presented in detail previously [3].

### Test validation

Considering that the ELISA kits used in the present study were similar to those used in our previous serosurvey, their diagnostic performance and test validation were the same as previously described [3]. Similarly, we used two scenarios to adjust the seroprevalence rates in this study. Scenario 1 test performance (our own data on tests validation, including the sensitivity of 66.9% and specificity of 98.2%) was used as the primary test characteristic, and scenario 2 (combining manufacture’s data with our data on tests validation, including the sensitivity of 71.8% and the specificity of 98.2%) was used to be compared with the scenario 1 test-adjusted estimates.

### Covariates

Demographic information included sex, age, and residence city. Past medical history included the following self-reported comorbidities: heart disease, hypertension, chronic lung disease, asthma, diabetes, obesity, and renal disease. COVID-19-related symptoms included cough, fever, chills, sore throat, headache, dyspnea, diarrhea, anosmia, conjunctivitis, weakness, myalgia, arthralgia, altered level of consciousness, and chest pain experienced over the past 12 weeks [3, 11]. Participants were then categorized as asymptomatic, paucisymptomatic (one to three symptoms), or symptomatic (four or more symptoms). We also asked participants about their recent contact (over the past 12 weeks) with a confirmed COVID-19 patient.

### Statistical analysis

The statistical analyses were previously explained in detail [3]. Briefly, the overall crude seroprevalence of SARS-CoV-2-specific antibodies was estimated as a proportion of the positive tests to the total sample size. Age-sex-city population-weighted rates were computed within bootstrap samples using the 2016 population and household census in Iran as the standard population. Given the nature of participant selection, the bootstrap weighted seroprevalence rate for each combination of cities (Ahvaz, Ardabil, Babol, Gorgan, Hamedan, Kerman, Kermanshah, Mashhad, Qom, Sanandaj, Sari, Shiraz, Tabriz, Tehran, Urmia, and Zahedan), age (10-19, 20-29, 30-39, 40-49, 50-59, ≥60) and sex (male, female) was performed. Finally, to minimize the resultant bias due to imperfect sensitivity and specificity antibody tests, we calculated the test performance adjusted of weighted seroprevalence (bootstrap weight) for scenarios 1 and 2 based on Cassaniti’s et al. [12, 13] proposed following formula, where AP denoted adjusted prevalence, UP denoted unadjusted prevalence (apparent prevalence), Sp denoted test specificity, and Se denoted test sensitivity:

$$\mathrm{AP}=\frac{\mathrm{UP}+\mathrm{Sp}-1}{\mathrm{Se}+\mathrm{Sp}-1}$$

It should be noted that 95% confidence intervals (CIs) for unweighted seroprevalence were estimated using exact binomial models, and a bootstrap method was used to construct the 95% CIs for weighted and adjusted estimates [14, 15]. Categorical variables were reported as frequency and percentage. We calculated the total number of infections by multiplying infection prevalence by the total population of each province. We also assessed the distribution of SARS-COV-2 seropositivity according to sex, age, comorbidity, contact with COVID-19 patients, and symptoms, using chi-squared test. All statistical analyses were performed using Microsoft Excel and STATA version 14 (StataCorp, College Station, TX).

## Results

**Fig. 2:**
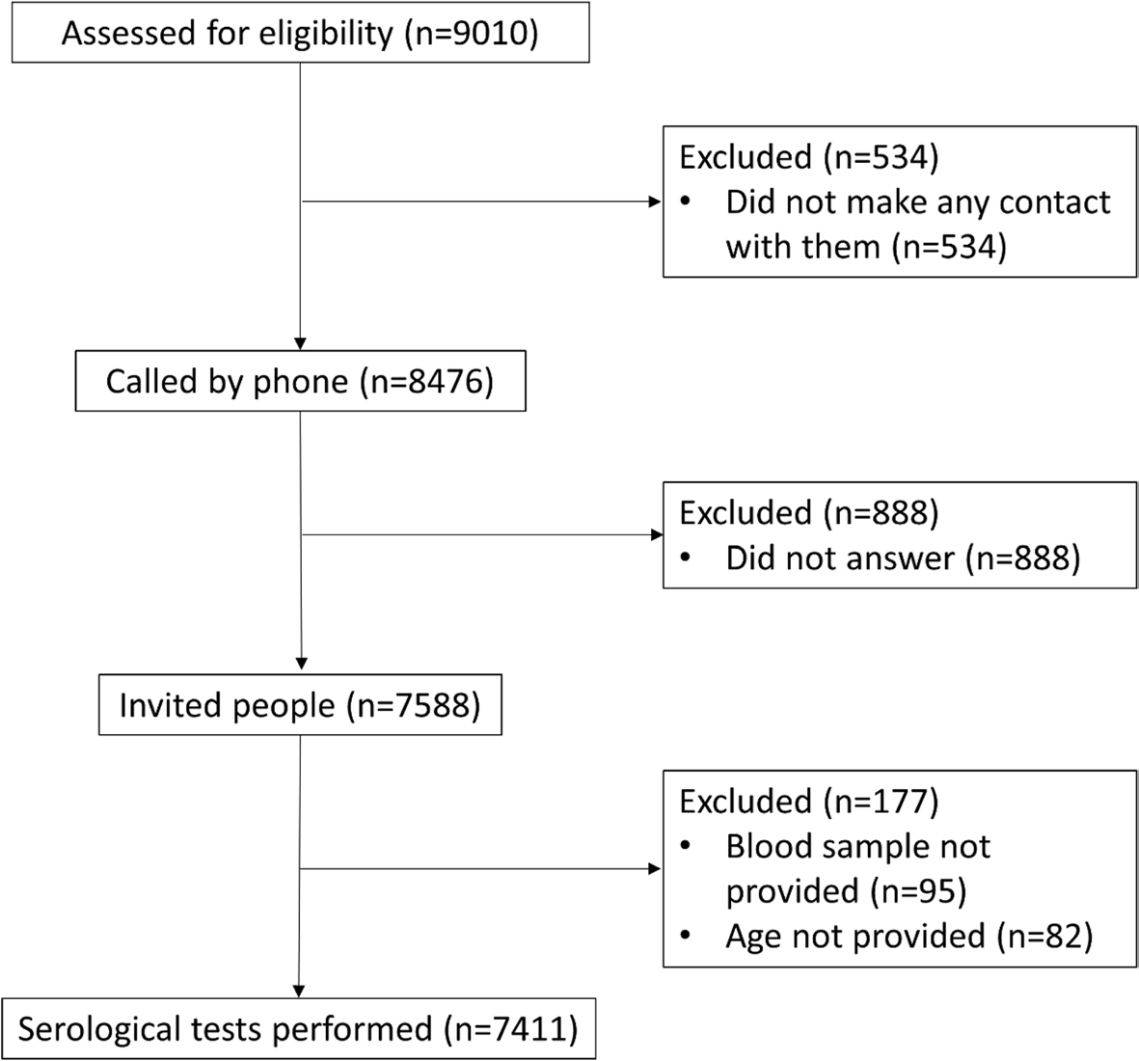
Flowchart of participants recruitment

Among 9010 individuals selected from 16 cities (total population 22,420,684) to participate in this study, 7411 (82.3%) individuals consented and were enrolled (Fig. 2). The data collection lasted from January to March, 2021. Supplemental Table 1 indicates the basic information of the participants by city. Out of 7411 individuals finally included in the analysis, 3721 (50.2%) were male, 2229 (30.3%) had at least one comorbidity, and 2557 (35.0%) reported recent contact with a confirmed COVID-19 patient. The mean age was 41.3 ± 13.0 years, ranging from 10 to 90 years old, and majority of the participants were aged 30-39 (*n* = 2202, 29.7%) years old. It should be mentioned that 56 (0.8%) and 59 (0.8%) participants did not complete the questions on COVID-19-related symptoms and comorbidity, respectively. The distribution of participants across the 16 cities has been denoted in the Supplemental Fig. 1.

A total of 1764 individuals tested positive for the presence of IgM or IgG antibodies against SARS-CoV-2, resulting in a crude seroprevalence of 23.8% (95% CI 22.8-24.8; Table 1). Across the cities, the crude seropositivity rate ranged from 14.5% (Ahvaz) to 37.1% (Tabriz) (Table 2).

**Table 1:** Seroprevalence of severe acute respiratory syndrome coronavirus 2-specifc IgG and IgM antibodies

	Sample size, N	Seropositive participants, n	Seroprevalence				
			Crude (95% CI)	Weighted (95% CI) ^a^	Adjusted for test Scenario I (95% CI)^b^	Adjusted for test Scenario II (95% CI)^c^	***P***_value
**Total**	7411	1764	23.8 (22.8-24.8)	24.0 (21.8-26.2)	34.2 (31.0-37.3)	31.8 (28.8-34.7)	
**Sex**							
**Male**	3721	876	23.5 (22.2-25.0)	23.7 (20.4-26.9)	33.6 (28.4-38.8)	31.2 (26.7-35.7)	0.597
**Female**	3690	888	24.1 (22.7-25.5)	24.3 (21.5-27.1)	34.7 (30.5-38.9)	32.3 (28.4-36.1)	
**Age, years**							
**10-19**	362	85	23.5 (19.2-28.2)	20.9 (14.7-27.2)	29.7 (20.1-39.4)	27.7 (18.7-36.6)	< 0.0001
**20-29**	879	183	20.8 (18.2-23.7)	20.9 (17.2-24.7)	29.4 (23.9-35.0)	27.4 (21.7-33.1)	
**30-39**	2202	480	21.8 (20.1-23.6)	23.1 (19.2-26.9)	32.6 (26.5-38.8)	30.4 (24.6-36.1)	
**40-49**	2051	492	24.0 (22.1-25.9)	23.4 (19.9-26.8)	33.2 (27.9-38.4)	30.8 (26.0-35.7)	
**50-59**	1272	331	26.0 (23.6-28.5)	25.0 (21.1-29.0)	35.7 (29.4-42.0)	33.2 (27.4-39.0)	
**≥ 60**	645	193	29.9 (26.4-33.6)	34.4 (28.1-40.6)	50.2 (40.7-59.7)	46.7 (38.5-54.9)	
**Comorbidity**							
**Yes**	2229	561	25.2 (23.4-27.0)	23.7 (21.5-25.8)	32.4 (25.4-39.4)	30.1 (23.8-36.5)	0.062
**No**	5123	1186	23.1 (22.0-24.3)	22.9 (18.3-27.5)	33.6 (30.3-37.0)	31.3 (28.1-34.4)	
**Contact with confirmed COVID-19 patients**							
**Yes**	2557	730	28.5 (26.8-30.3)	29.5 (23.1-35.9)	42.6 (32.8-52.4)	37.0 (28.5-45.4)	< 0.0001
**No**	4739	1010	21.3 (20.1-22.5)	21.3 (19.6-23.1)	30.0 (27.3-32.8)	26.1 (23.7-28.4)	
**Symptoms**							
Asymptomatic (0)	4409	888	20.1 (19.0-21.3)	20.86 (18.86-22.85)	29.0 (26.2-31.9)	27.0 (24.6-29.5)	< 0.0001
Paucisymptomatic (1-3)	1797	398	22.1 (20.2-24.1)	21.77 (17.10-26.44)	32.9 (26.2-39.7)	30.6 (24.6-36.7)	
Symptomatic (≥4)	1149	460	40.0 (37.2-42.9)	37.5 (28.68-46.32)	61.0 (47.2-74.7)	56.8 (44.2-69.3)	

Seroprevalence data are % (95% confidence interval). ^a^Weighted for age, sex and city population. ^b^Weighted prevalence adjusted for test performance I (sensitivity 66·9% and specificity 98.2%). ^c^Weighted prevalence adjusted for test performance II as reported by manufacturer (sensitivity 71.8% and specificity 98.2%). When a variable was stratified it was removed from the weight

**Table 2:** Seroprevalence of SARS-COV-2 in first (general population) and second phases by city

City	Phases	Number, n/N	Crude (95% CI)	Weighted (95% CI)^a^	Adjusted for test performance I (95% CI)^b^	Adjusted for test performance II (95% CI)^c^
**Ahvaz**	First phase	8/100	8.0 (3.5-15.5)	7.9 (2.2-15.9)	9.4 (0.7-21.6)	8.7 (0.6-20.1)
	Second phase	57/393	14.5 (11.2-18.4)	17.7 (5.1-30.3)	20.4 (0.5-40.2)	18.9 (0.6-37.2)
**Ardabil**	First phase	11/87	12.6 (6.5-21.5)	14.8 (3.9-32.3)	20.0 (3.2-46.8)	18.6 (3.0-43.5)
	Second phase	115/384	29.9 (25.4-34.8)	28.8 (22.8-34.8)	34.1 (24.2-44.0)	31.7 (22.7-40.7)
**Babol**	First phase	19/91	20.9 (13.1-30.7)	16.4 (9.6-24.6)	22.4 (11.9-35.1)	20.8 (11.1-32.6)
	Second phase	100/383	26.1 (21.8-30.8)	23.1 (14.3-31.8)	27.0 (16.6-37.4)	25.1 (15.3-34.9)
**Gorgan**	First phase	41/125	32.8 (24.7-41.8)	30.4 (22.3-39.7)	43.9 (31.4-58.3)	40.9 (29.2-54.2)
	Second phase	62/330	18.8 (14.7-23.4)	22.4 (10.7-34.1)	26.1 (6.9-45.4)	24.3 (6.9-41.7)
**Hamedan**	First phase	8/108	7.4 (3.5-15.2)	7.2 (2.9-12.9)	8.3 (1.6-17.0)	7.7 (1.5-15.8)
	Second phase	116/391	29.7 (25.2-34.5)	27.5 (21.7-33.3)	32.4 (22.3-42.6)	30.1 (20.7-39.6)
**Kerman**	First phase	10/108	9.3 (4.5-16.4)	7.1 (3.2-12.6)	8.2 (2.2-16.6)	7.7 (2.1-15.4)
	Second phase	64/355	18.0 (14.2-22.4)	14.5 (9.3-19.7)	16.0 (10.7-21.4)	14.9 (9.8-20.0)
**Kermanshah**	First phase	14/133	10.6 (5.9-17.1)	13.1 (5.3-21.9)	17.3 (5.3-30.9)	16.1 (4.9-28.8)
	Second phase	92/389	23.6 (19.5-28.2)	24.1 (17.4-30.7)	28.3 (15.9-40.7)	26.3 (14.5-38.1)
**Mashhad**	First phase	21/176	11.9 (7.5-17.6)	11.5 (7.1-16.8)	14.8 (8.2-23.1)	13.8 (7.6-21.5)
	Second phase	171/691	24.7 (21.6-28.1)	24.7 (17.7-31.7)	35.2 (23.6-46.8)	32.7 (21.2-44.2)
**Qom**	First phase	48/108	44.4 (34.8-54.3)	39.9 (26.0-56.4)	58.5 (37.2-83.9)	54.4 (34.6-78.0)
	Second phase	114/385	29.6 (25.1-34.4)	27.2 (18.1-36.2)	29.8 (24.0-35.6)	32.0 (25.9-38.2)
**Sanandaj**	First phase	4/96	4.2 (1.1-10.3)	2.7 (0.6-5.7)	1.7 (0.0-6.0)	1.6 (0.0-5.6)
	Second phase	117/388	30.1 (25.6-35.0)	27.9 (22.2-33.6)	33.0 (27.3-38.6)	30.7 (25.3-36.1)
**Sari**	First phase	22/175	12.6 (8.0-18.4)	11.4 (6.9-16.4)	14.7 (7.8-22.4)	13.7 (7.3-20.8)
	Second phase	74/400	18.5 (14.8-22.7)	15.9 (9.0-22.7)	17.8 (11.0-24.5)	16.5 (9.8-23.2)
**Shiraz**	First phase	10/124	8.1 (3.9-15.0)	6.6 (2.6-11.20	7.3 (1.2-14.5)	6.8 (1.1-13.5)
	Second phase	90/485	18.6 (15.2-22.3)	18.1 (13.1-23.2)	25.4 (13.6-37.1)	23.6 (12.5-34.7)
**Tabriz**	First phase	8/103	7.8 (3.4-14.7)	5.4 (1.7-10.2)	5.6 (0.0-13.0)	5.2 (0.0-12.1)
	Second phase	180/485	37.1 (32.8-41.6)	32.8 (24.7-40.8)	39.2 (33.0-45.5)	36.5 (30.8-42.2)
**Tehran**	First phase	191/1572	12.1 (10.6-13.9)	12.4 (10.6-14.5)	16.3 (13.5-19.5)	15.1 (12.5-18.2)
	Second phase	271/1181	22.9 (20.6-25.4)	24.9 (20.0-29.8)	35.5 (27.1-43.9)	33.0 (25.0-41.0)
**Urmia**	First phase	8/101	7.9 (3.5-15.0)	8.4 (1.9-17.6)	10.0 (0.2-24.3)	9.3 (0.2-22.6)
	Second phase	66/389	17.0 (13.4-21.1)	18.4 (12.4-24.3)	20.8 (9.0-32.5)	19.3 (8.2-30.4)
**Zahedan**	First phase	8/105	7.6 (3.3-14.5)	9.7 (3.3-16.9)	12.1 (2.3-23.3)	11.3 (2.1-21.6)
	Second phase	75/382	19.6 (15.8-24.0)	16.2 (9.8-22.6)	18.0 (7.6-28.4)	16.7 (7.3-26.1)

Seroprevalence data are % (95% confidence interval). ^a^Weighted by age and sex for each city population·^b^Weighted prevalence adjusted for test performance I (sensitivity 66·9% and specificity 98.2%). ^c^Weighted prevalence adjusted for test performance II as reported by manufacturer (sensitivity 71.8% and specificity 98.2%). Sample sizes of the first and second phases were 3530 and 7411, respectively

The overall population-weighted seroprevalence adjusted for test performance was 34.2% (95% CI 31.0-37.3; Table 1), with an estimated 7,667,874 (95% CI 6,950,412-8,362,915) infections from the 16 cities. This rate was lowest among individuals aged 20-29 years (29.4% [95% CI 23.9-35.0]) and highest among those aged ≥60 years (50.2% [95% CI 40.7-59.7]), and appeared to be lower in males (33.6% [95% CI 28.4-38.8]) than in females (34.7% [95% CI 30.5-38.9]). The seroprevalence was 32.4% (95% CI 25.4-39.4) in the people with at least one comorbidity, versus 33.6% (95% CI 30.3-37.0) in those without. Moreover, individuals with a contact with a confirmed COVID-19 patient had a higher seroprevalence rate than those without (42.6% [95% CI 32.8-52.4] versus 30.0% [95% CI 27.3-32.8]). Seroprevalence of SARS-COV-2 by antibody positivity was represented in Supplemental Table 2.

Across the cities, the highest population weight-adjusted and test-adjusted prevalence rates of SARS-CoV-2 were related to Tabriz (39.2%), Tehran (35.5%), and Mashhad (35.2%), while the lowest estimates were related to Kerman (16.0%), Sari (17.8%) and Zahedan (18.0%). The results of the first and second serosurveys in Iran have been represented in Table 2 and Supplemental Fig. 2. Comparing the results showed that most of the cities reported an increased number of infected cases between April, 2020, and March, 2021, except Qom and Gorgan, which were associated with a decrease in the seroprevalence over the same period.

The most common symptoms reported were headache (20.4%), sore throat (16.0%), weakness (14.9%), and cough (14.3%) (Supplemental Table 3). The test-adjusted seroprevalence of COVID-19 was 61.0% (95% CI 47.2-74.7) in symptomatic individuals, which was higher than those in paucisymptomatic (32.9% [95% CI 26.2-39.7]) and asymptomatic (29.0% [95% CI 26.2-31.9]) individuals (Supplemental Table 4). Among 1746 seropositive individuals, 858 (49.1%) reported a history of symptoms suggestive of COVID-19, but 888 (50.9%) reported no symptoms, implying that an estimated number of 3,902,948 (95% CI 3,537,760-4,256,724) individuals infected by March, 2021, were asymptomatic in the total populations of the 16 cities studied (Supplemental Fig. 3 and Table 4). Finally, COVID-19-related symptoms were observed more frequently in the participants aged 30-39 or 40-49 years old than in other age groups (Supplemental Fig. 4).

We observed lower estimates for the scenario 2 test-adjusted seroprevalence in comparison with the scenario 1 test-adjusted estimates, which is mainly attributed to the higher sensitivity in the scenario 2 test performance; however, there was a consistency between the two test performance scenarios in the trends seen for the seroprevalence estimates in all analyses.

## Discussion

This study was the second large population-based study to determine the seroprevalence status of SARS-CoV-2 infection among the general population in 16 cities in Iran. It was demonstrated that approximately 34% of the study population aged 10 years or older had been exposed to SARS-CoV-2 infection by March, 2021, implying an estimation of 7.6 million infections that is much higher than the number of confirmed COVID-19 cases officially reported throughout the country at the same time (nearly 1.9 million infections) [16]. This inconsistency could reflect the fact that cases are mainly diagnosed in the symptomatic phases of the disease; for example, a serosurvey from the USA reported that the estimated number of SARS-CoV-2 infections was 6 to 24 times more than the number of officially reported cases [17]. Variable clinical management of mild cases and false-negative results of virological tests could be other potential reasons for the discrepancy.

Comparing with the previous large serosurvey in Iran, the total seroprevalence increased by about two times among the general population, from 17.1% in April, 2020, to 34.2% in March, 2021 [3]. One of the advantages of the present study compared with the previous one was a larger sample size of the general population included (7411 versus 3530 individuals). Furthermore, we tried to focus only on the general population in the second phase, while both of the general population and high-risk occupational groups were studied in the first survey.

The rise in the seroprevalence was observed in most of the cities studied. Tabriz and Sanandaj cities were associated with the greatest increase in the seroprevalence (by 33.6 and 31.3%, respectively). Prolonged COVID-19-related social restrictions, which could potentially influence population mental health, as well as insufficient awareness about the importance of the disease, could apparently have had negative effects on the people’s compliance with Infection Prevention and Control (IPC), and could potentially have been the main reasons for the increased rate of SARS-CoV-2 seropositivity in these areas. Compliance with IPC protocols has an important role in minimizing the risk of SARS-CoV-2 infection, as it has been shown that use of face masks and physical distancing could increase the probability of the COVID-19 transmission control [18]. In addition to these, the emergence of new genetic variants of SARS-CoV-2 could partially explain the increased rate of seroprevalence; for instance, alpha (B.1.1.7) variant, which is more transmissible than the previous wildtype lineage [19], was predominantly circulating in Iran at the time of this study [20], potentially leading to speed up the viral spread and, consequently, more infections. The rise in the seroprevalence demonstrates the widespread infection in the abovementioned cities as well.

On the other hand, compared with previous study performed during the first epidemic wave [3], Qom and Gorgan cities reported reduced numbers of infected cases among general population between April, 2020, and March, 2021. It is noteworthy that these regions were of the first districts reported increased number of COVID-19 cases early in the epidemic [3, 21], as also shown in the previous study, both cities had relatively high seropositivity rates compared with other cities among general population (Qom, 58.5%; Gorgan, 43.9%); therefore, more stringent observance of the IPC protocols could be expected from the people of those two cities during the second and third waves, leading to better controlling the spread of the virus, although no evidence exists to support this supposition yet. Finally, another possible reason for this reduction might relate to waning of antibodies.

Our findings also indicated that the seroprevalence did not differ by sex, while it rose with an increase in age. These findings were compatible with our previous large serosurvey in Iran. Other studies reported variable results on the age-specific seroprevalence pattern of SARS-CoV-2; some were in agreement with our findings [22, 23], while others were not [24, 25]. A higher seroprevalence in older age groups versus younger ages potentially reflects the more severe nature of the disease in the elderly [26]. Another possible reason could be a higher waning of antibodies in younger age groups compared with older ages [27].

To the best of our knowledge, no similar serosurveys have been done in the same period as our study in Iran (after the third wave) either at the national or regional level, and all available studies pertains to previous waves [9, 28–31]; hence, we are not able to compare our results with any Iranian studies. The seroprevalence rate estimated in the present study (34.2%) was higher than estimates from the USA, such as Georgia (8.6% [weighted seroprevalence]) [32], and Cincinnati Ohio (12.9% [unweighted seroprevalence]) [33], Denmark (4.0% [test-performance adjusted seroprevalence]) [34], India (24.1% [weighted and test-performance adjusted seroprevalence]) [22], Sierra Leone (2.6% [weighted seroprevalence]) [35], and South Africa (27% [test-performance adjusted seroprevalence]) [36], which could be partly attributed to the fact that the onset of the COVID-19 epidemic in Iran was earlier than the given countries, leading to longer exposure of Iranian population to the virus and a higher risk of the infection. Furthermore, differences in the IPC protocols and their observance, as well as the social and climatic conditions, in each country could be other causes of the discrepancy in the seroprevalence rate. Of course, it should be mentioned that the abovementioned studies from the USA and Denmark were conducted during the surge of COVID-19 in these countries and we witnessed a decreasing trend in the disease thereafter [1]; however, the SARS-CoV-2 seroprevalence was considerably still higher in our country than in those countries.

The COVID-19 vaccination was initiated in February, 2021 in Iran [37]. However, it should be mentioned that during the present study (between January to March, 2021), the healthcare workers were only vaccinated, but not other populations; therefore, none of the individuals included in this study were vaccinated, and therefore, response measures to COVID-19 should not be affected.

We did not enroll the high-risk populations in this study because of two reasons. First, we did not find a significant difference between the high-risk and general populations in the COVID-19 seroprevalence in the first study phase; therefore, we found it unnecessary to assess the high-risk population in this study. Second, healthcare workers were vaccinated during the period of the present study, potentially affecting the seroprevalence.

This study has also some limitations. First, in Tehran and Mashhad cities, we could not enroll individuals from SIB network because of a very low response rate during the strict lockdowns; therefore, we selected the sample from the employee cohorts of the Iran University of Medical Sciences and Mashhad University of Medical Sciences for these two cities [38]. Considering that the aforementioned cohorts were conducted on the general population, selection bias is expected to be partially controlled; however, the estimates for those two cities should be interpreted with caution. Overall, the non-response rate in this study was higher than what we primarily assumed; it should also be stated that despite our attempts, we, unfortunately, could not collect any data regarding the study outcome from the non-responders in any centers; therefore, we were not able to evaluate the distribution of the data by region, age, and/or gender. We alluded to this point in the limitations Second, the study sampling was restricted to only urban areas, and rural areas were not included. Third, the sensitivity of ELISA tests was lower in our study than that reported in other countries [39]; however, to overcome this issue, we reassessed the diagnostic accuracy of the assays and adjusted the estimates of the COVID-19 seroprevalence for the test performance. Finally, vanishing antibodies over time and the resultant negative serological testing in some people could probably underestimate the SARS-CoV-2 seroprevalence rates.

## Conclusions

The results of the present study showed that nearly one in three individuals aged ≥10 years old were exposed to SARS-CoV-2 in the cities studied by March, 2021. The seroprevalence increased by about two times between April, 2020, and March, 2021. Moreover, the seropositivity was much higher than the number of confirmed COVID-19 cases officially reported. In addition to the assessment of the disease burden, our surveillance would be helpful to identify high-risk areas to target interventions, to monitor the trends and detect outbreaks (for guiding the public health practice), and to monitor the levels of immunity within different age groups. Since the infection rate is increasing in Iran, and a large proportion of the population is still susceptible, it is important to continue implementing the protocols of infection prevention and control. As of May 6, 2022, more than 68% of the Iranian population have been fully vaccinated [37]; therefore, the vaccination needs to be performed faster to make it possible to overcome the epidemic. Finally, further phases of the population-based serosurvey are recommended to continue reporting the latest status of the SARS-CoV-2 epidemiology.

## Supplementary Information

**Additional file 1.**

## Data Availability

The datasets generated and/or analysed during the current study are not publicly available due to ethical restrictions, but are available from the corresponding author on reasonable request.

## Abbreviations

COVID-19: Coronavirus disease 2019
SARS-CoV-2: Severe acute respiratory syndrome coronavirus 2
ELISA: Enzyme-linked immunosorbent assay
CI: Confidence interval
IPC: Infection Prevention and Control
