# Supplementary material for: Seroprevalence of SARS-CoV-2 antibody among urban Iranian population: findings from the second large population-based cross-sectional study
Source: BMC Public Health. 2022 May 23;22:1031. doi: 10.1186/s12889-022-13464-7 (PMC9125542; doi:10.1186/s12889-022-13464-7)
Supplement: Supplementary file 1 — Additional file 1. [file 12889_2022_13464_MOESM1_ESM.pdf]

Supplementary Materials

Seroprevalence of SARS-CoV-2 antibody among urban Iranian population: findings from the second large population-based cross-sectional study

Mohammad Zamani[1*], Hossein Poustchi[2*], Zahra Mohammadi[2], Sahar Dalvand[2], Maryam Sharafkhah[2], Seyed Abbas Motevalian[3,4], Saeid Eslami[5], Amir Emami[6], Mohammad Hossein Somi[7], Jamshid Yazdani-Charati[8], Nader Saki[9], Manoochehr Karami[10,11], Farid Najafi[12], Iraj Mohebbi[13], Nasrollah Veisi[14], Ahmad Hormati[15,16], Farhad Pourfarzi[17], Reza Ghadimi[18], Alireza Ansari-Moghaddam[19], Hamid Sharifi[20], Gholamreza Roshandel[21], Fariborz Mansour-Ghanaei[22], Farahnaz Joukar[23], Amaneh Shayanrad[2], Sareh Eghtesad[2], Ahmadreza Niavarani[24], Alireza Delavari[24], Soudeh Kaveh[2], Akbar Feizesani[2], Melineh Markarian[1], Fatemeh Shafighian[24], Alireza Sadjadi[24], Maryam Darvishian[25], Reza Malekzadeh[24,1]

* Denotes joint first author

1. Digestive Diseases Research Center, Digestive Diseases Research Institute, Shariati Hospital, Tehran University of Medical Sciences, Tehran, Iran.
2. Liver and Pancreatobiliary Diseases Research Center, Digestive Diseases Research Institute, Shariati Hospital, Tehran University of Medical Sciences, Tehran, Iran.
3. Research Center for Addiction and Risky Behaviors (ReCARB), Psychosocial Health Research Institute, Iran University of Medical Sciences, Tehran, Iran.
4. Department of Epidemiology, School of Public Health, Iran University of Medical Sciences, Tehran, Iran.
5. Pharmaceutical Research Center, Pharmaceutical Technology Institute, Mashhad University of Medical Sciences, Mashhad, Iran.
6. Microbiology Department, Burn & Wound Healing Research Center, Shiraz University of Medical Sciences, Shiraz, Iran.

7. Liver and Gastrointestinal Diseases Research Center, Tabriz University of Medical Sciences, Tabriz, Iran.
8. Department of Biostatistics, Health Sciences Research Center, Addiction Institute, Faculty of Health, Mazandaran University of Medical Sciences, Sari, Iran.
9. Hearing Research Center, Ahvaz Jundishapur University of Medical Sciences, Ahvaz, Iran.
10. Department of Epidemiology, School of Public Health, Hamadan University of Medical Sciences, Hamadan, Iran.
11. Department of Epidemiology, School of Public Health & Safety, Shahid Beheshti University of Medical Sciences, Tehran, Iran.
12. Research Center for Environmental Determinants of Health, School of Public Health, Kermanshah UMS, Kermanshah, Iran.
13. Social Determinants of Health Center, Urmia University of Medical Sciences, Urmia, Iran.
14. Kurdistan University of Medical Sciences, Sanandaj, Iran.
15. Gastroenterology and Hepatology Disease Research Center, Qom University of Medical Science, Qom, Iran.
16. Department of Internal Medicine, School of Medicine, Gastrointestinal and Liver Diseases Research Center, Iran University of Medical Sciences, Tehran, Iran.
17. Digestive Disease Research Center, Ardabil University of Medical Sciences, Ardabil, Iran.
18. Social Determinants of Health Research Center, Health Research Institute, Babol University of Medical Sciences, Babol, Iran.
19. Health Promotion Research Center, Zahedan University of Medical Sciences, Zahedan, Iran.
20. HIV/STI Surveillance Research Center and WHO Collaborating Center for HIV Surveillance, Institute for Futures Studies in Health, Kerman University of Medical Sciences, Kerman, Iran.
21. Golestan Research Center of Gastroenterology and Hepatology, Golestan University of Medical Sciences, Gorgan, Iran.
22. Division of Gastroenterology & Hepatology, Gastrointestinal & Liver Diseases Research Center, Guilan University of Medical Sciences, Rasht, Iran.
23. Gastrointestinal and Liver Diseases Research Center, Guilan University of Medical Sciences, Rasht, Iran.
24. Digestive Oncology Research Center, Digestive Diseases Research Institute, Shariati Hospital, Tehran University of Medical Sciences, Tehran, Iran.

25. Cancer Control Research, BC Cancer Research Centre, Vancouver, BC, Canada.

Correspondence to:

Prof Reza Malekzadeh,

Digestive Diseases Research Institute, Shariati Hospital, Tehran University of Medical Sciences, Tehran 14117–13135, Iran

Email: dr.reza.malekzadeh@gmail.com

Phone: +98 21 824 15 000

Table S1. Baseline characteristics of study participants by city

	Population size		Sex	Age (Year)						Comorbidity condition		Contact with COVID-19 patients	
	Total	Sample	Male	10-19	20-29	30-39	40-49	50-59	≥60	No	Yes (≥1)	No	Yes
**Overall**	22420684	7411	3721	362	879	2202	2051	1272	645	5123	2229	4739	2557
			(50.2)	(4.8)	(11.9)	(29.7)	(27.7)	(17.2)	(8.7)	(69.7)	(30.3)	(65.0)	(35.0)
**Ahvaz**	1184788	393	166	6	44	160	111	51	21	248/393	145/393	246/392	146/392
			(42.2)	(1.5)	(11.2)	(40.7)	(28.2)	(13.0)	(5.3)	(63.1)	(36.9)	(62.8)	(37.2)
**Ardabil**	529374	384	197	26	54	108	107	66	23	285/384	99/384	294/383	89/383
			(51.3)	(6.8)	(14.1)	(28.1)	(27.9)	(17.2)	(6.0)	(74.2)	(25.8)	(76.8)	(23.2)
**Babol**	250217	383	184	13	43	82	95	86	64	242/382	140/382	248/379	131/379
			(48)	(3.4)	(11.2)	(21.4)	(24.8)	(22.5)	(16.7)	(63.4)	(36.6)	(65.4)	(34.6)
**Gorgan**	350676	330	71	14	30	101	119	47	19	246/330	84/330	251/329	78/329
			(21.5)	(4.2)	(9.1)	(30.6)	(36.1)	(14.2)	(5.8)	(74.5)	(25.5)	(76.3)	(23.7)
**Hamedan**	554406	391	234	15	55	89	117	67	48	293/391	98/391	254/390	136/390
			(59.8)	(3.8)	(14.1)	(22.8)	(29.9)	(17.1)	(12.3)	(74.9)	(25.1)	(65.1)	(34.9)
**Kerman**	537718	355	191	14	53	132	81	45	30	266/352	86/352	250/318	68/318
			(53.8)	(3.9)	(14.9)	(37.2)	(22.8)	(12.7)	(8.5)	(75.6)	(24.4)	(78.6)	(21.4)
**Kermanshah**	946651	389	203	90	61	89	69	51	29	324/386	62/386	317/386	69/386
			(52.2)	(23.1)	(15.7)	(22.9)	(17.7)	(13.1)	(7.5)	(83.9)	(16.1)	(82.1)	(17.9)
**Mashhad**	3001184	691	338	0	97	229	222	124	19	464/649	185/649	333/639	306/639

			(48.9)	(0.0)	(14.0)	(33.1)	(32.1)	(17.9)	(2.7)	(71.5)	(28.5)	(52.1)	(47.9)
**Qom**	1201158	385	123	20	70	140	99	39	17	294/381	87/381	214/379	165/379
			(31.9)	(5.2)	(18.2)	(36.4)	(25.7)	(10.1)	(4.4)	(77.2)	(22.8)	(56.5)	(43.5)
**Sanandaj**	412767	388	209	57	58	112	75	48	38	249/388	139/388	253/387	134/387
			(53.9)	(14.7)	(14.9)	(28.9)	(19.3)	(12.4)	(9.8)	(64.2)	(35.8)	(65.4)	(34.6)
**Sari**	309820	400	232	9	44	109	113	73	52	343/400	136/400	239/399	160/399
			(58.0)	(2.3)	(11.0)	(27.3)	(28.2)	(18.3)	(13.0)	(71.6)	(28.4)	(59.9)	(40.1)
**Shiraz**	1565572	485	287	0	63	218	134	57	13	343/479	136/479	154/481	327/481
			(59.2)	(0.0)	(13.0)	(44.9)	(27.6)	(11.8)	(2.7)	(71.6)	(28.4)	(32.0)	(68.0)
**Tabriz**	1558693	485	223	11	52	165	127	81	49	312/485	173/485	272/485	213/485
			(46.0)	(2.3)	(10.7)	(34.0)	(26.2)	(16.7)	(10.1)	(64.3)	(35.7)	(56.1)	(43.9)
**Tehran**	8693706	1181	664	0	58	300	429	317	77	795/1181	386/1181	792/1180	388/1180
			(56.2)	(0.0)	(4.9)	(25.4)	(36.3)	(26.8)	(6.5)	(67.3)	(32.7)	(67.1)	(32.9)
**Urmia**	736224	389	200	44	45	94	93	71	42	283/389	106/389	300/387	87/387
			(51.4)	(11.3)	(11.6)	(24.2)	(23.9)	(18.3)	(10.8)	(72.8)	(27.2)	(77.5)	(22.5)
**Zahedan**	587730	382	199	43	52	74	60	49	104	220/382	162/382	322/382	60/382
			(52.1)	(11.3)	(13.6)	(19.4)	(15.7)	(12.8)	(27.2)	(57.6)	(42.4)	(84.3)	(15.7)

Data are n, n (%), or n/N (%)

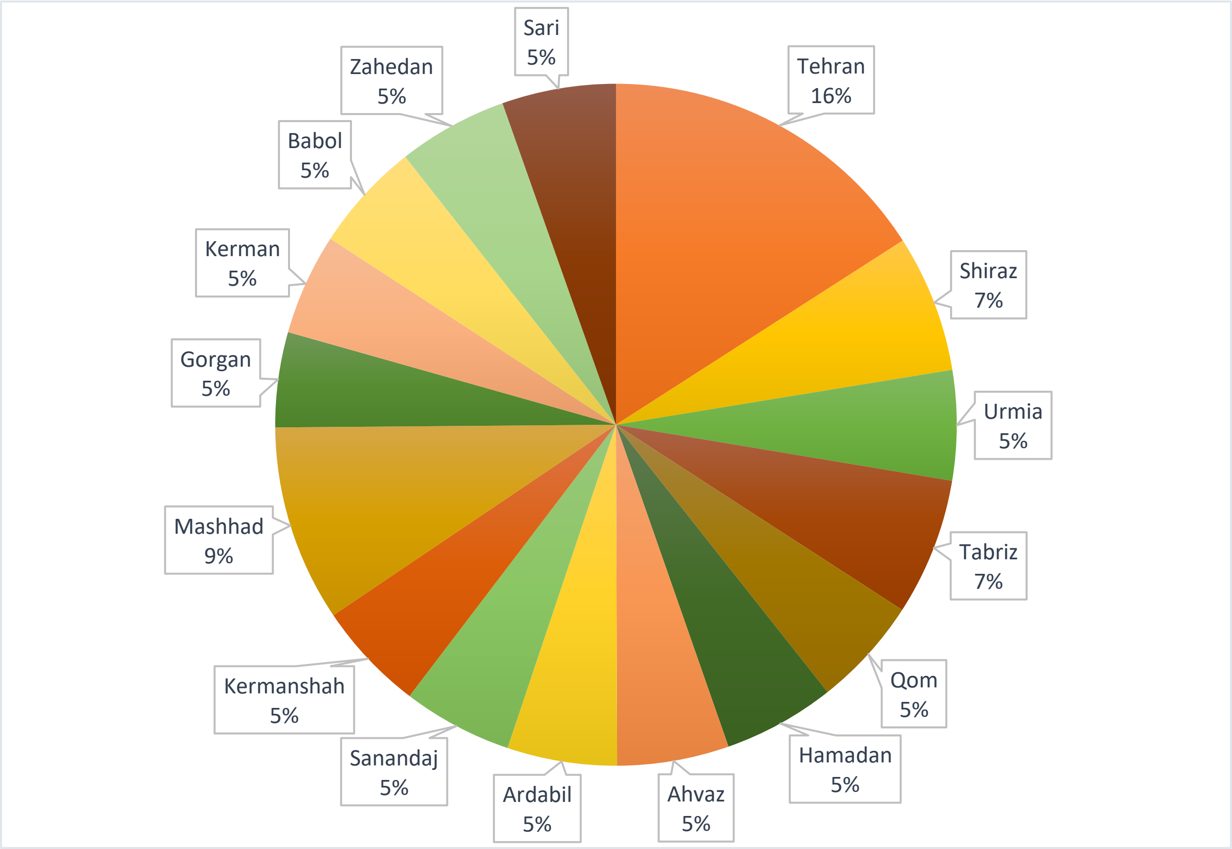

Figure S1. Distribution of participants across 16 cities studied.

First phase	Second phase

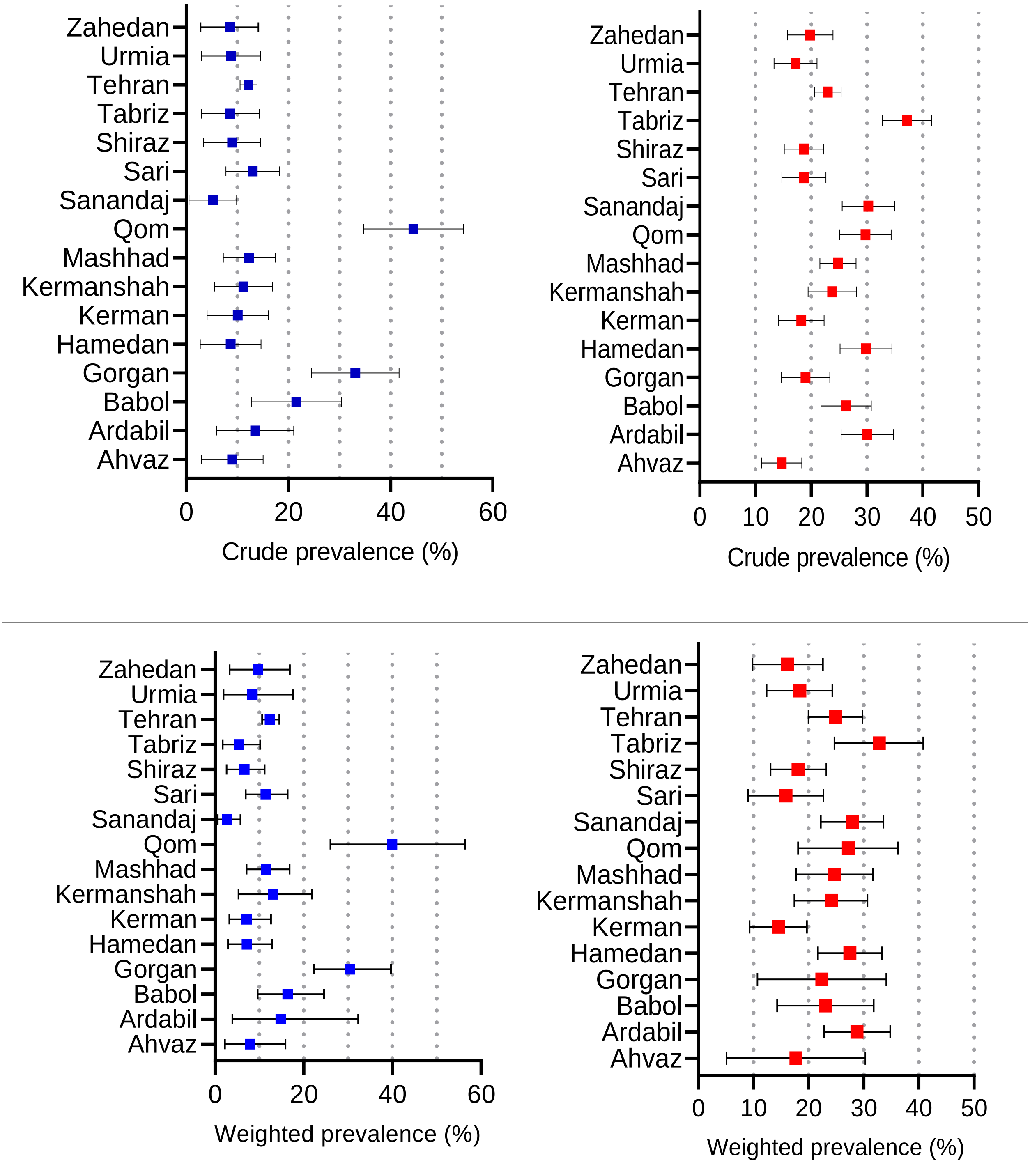

First phase	Second phase

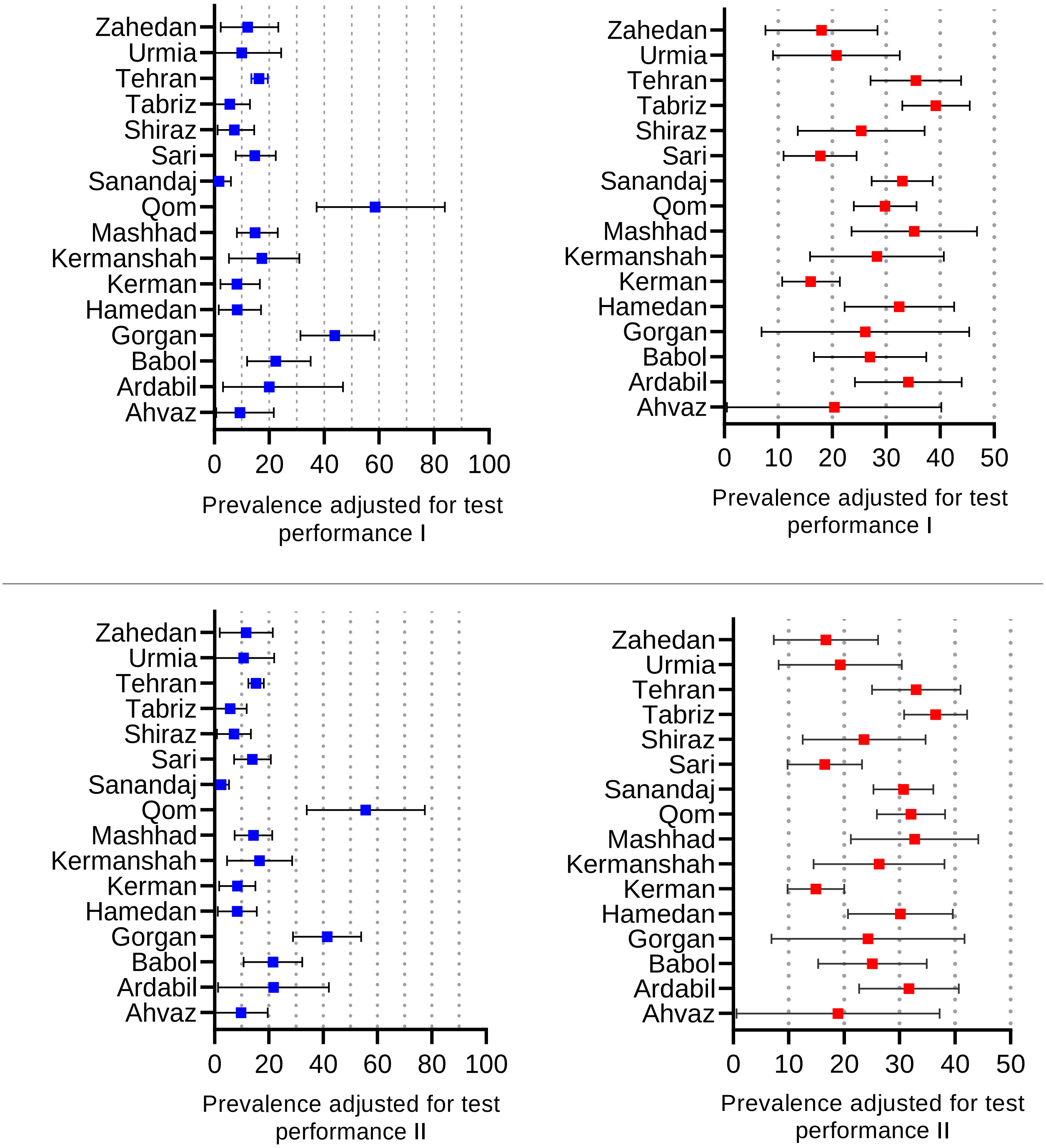

Figure S2. The crude (unadjusted) prevalence (95% CI), weighted prevalence, adjusted and weighted prevalence for test performance I (sensitivity 66·9% and specificity 98.2%), and adjusted and weighted prevalence for test performance II (sensitivity 71.8% and specificity 98.2%) of first and second phases studies of coronavirus disease in 16 cities of Iran by March 2021.

Table S2. Seroprevalence of SARS-COV-2 by antibody positivity.

	Number	Crude (95% CI)	Weighted (95% CI)	Adjusted for test performance I (95% CI)	Adjusted for test performance I (95% CI)
**IgG positive**	1570/7411	21.2 (20.3-22.1)	21.2 (19.0-23.3)	29.9 (26.5-33.3)	27.8 (24.7-31.0)
**IgM positive**	509/7411	6.9 (6.3-7.5)	6.4 (5.0-7.8)	7.6 (5.6-9.6)	7.0 (5.1-9.0)
**IgG or IgM positive**	1764/7411	23.8 (22.8-24.8)	24.0 (21.9-26.1)	34.2 (30.9-37.4)	31.8 (28.6-34.9)

Table S3. Seroprevalence of SARS-COV-2 among individuals with and without symptoms.

Frequency (%)	Status	Frequency (%)	Prevalence of antibody positive (95% CI)	Adjusted prevalence for test performance I (95% CI)†	Adjusted prevalence for test performance II (95% CI)‡
**Cough**	No	6301 (85.7)	21.5 (20.5-22.5)	31.1 (28.2-33.9)	28.9 (26.3-31.5)
	Yes	1054 (14.3)	37.2 (34.3-40.2)	55.3 (40.1-70.4)	52.0 (36.5-67.4)
**Fever**	No	6569 (89.3)	21.4 (20.4-22.4)	31.1 (27.9-34.3)	28.9 (26.0-31.8)
	Yes	786 (10.7)	43.4 (39.9-46.9)	62.7 (50.2-75.1)	58.3 (46.3-70.2)
**Chills**	No	6686 (90.9)	21.7 (20.8-22.7)	31.8 (28.5-35.1)	29.5 (26.3-32.7)
	Yes	669 (9.1)	43.6 (39.8-47.5)	62.4 (48.2-76.6)	58.6 (44.7-72.5)
**Anosmia**	No	6769 (92.0)	21.1 (20.2-22.1)	30.9 (27.1-34.6)	28.7 (25.2-32.2)
	Yes	586 (8.0)	53.7 (49.6-57.8)	75.5 (61.3-89.8)	72.0 (57.9-86.1)
**Sore throat**	No	6176 (84.0)	22.8 (21.7-23.8)	32.9 (29.6-36.1)	30.6 (27.4-33.7)
	Yes	1179 (16.0)	28.8 (26.3-31.5)	44.9 (36.1-53.8)	41.8 (33.5-50.0)
**Headache**	No	5857 (79.6)	22.5 (21.4-23.5)	32.2 (29.2-35.1)	29.9 (27.2-32.6)
	Yes	1498 (20.4)	28.8 (26.5-31.1)	47.3 (34.6-60.0)	44.0 (32.3-55.8)
**Dyspnea**	No	6855 (93.2)	22.5 (21.5-23.5)	32.8 (29.6-36.0)	30.5 (27.4-33.6)
	Yes	500 (6.8)	40.6 (36.3-45.0)	55.7 (41.3-70.2)	51.8 (38.6-65.0)
**Diarrhea**	No	6834 (92.9)	22.8 (21.8-23.8)	33.3 (29.9-36.7)	31.0 (28.0-33.9)
	Yes	521 (7.1)	36.1 (31.9-40.4)	53.2 (36.6-69.8)	50.0 (34.7-65.4)
**Conjunctivitis**	No	7051 (95.9)	23.3 (22.4-24.3)	33.8 (30.2-37.4)	31.5 (27.9-35.0)
	Yes	304 (4.1)	32.9 (27.6-38.5)	50.0 (36.5-63.5)	46.5 (34.1-58.8)
**Weakness**	No	6256 (85.1)	21.2 (20.2-22.3)	30.8 (27.9-33.7)	28.7 (26.0-31.4)
	Yes	1099 (14.9)	37.9 (35.1-40.9)	55.9 (40.8-71.0)	52.0 (38.2-65.8)
**Myalgia**	No	6343 (86.2)	21.4 (20.4-22.5)	31.2 (28.0-34.4)	29.0 (25.9-32.2)
	Yes	1012 (13.8)	38.1 (35.1-41.2)	55.0 (45.7-64.4)	51.2 (42.8-59.5)
**Arthralgia**	No	6822 (92.8)	22.3 (21.3-23.3)	32.3 (29.1-35.5)	30.1 (27.1-33.0)
	Yes	533 (7.2)	42.8 (38.5-47.1)	60.0 (46.5-73.6)	55.8 (43.5-68.1)
**Altered level of consciousness**	No	7126 (96.9)	23.4 (22.4-24.4)	33.9 (30.6-37.2)	31.5 (28.5-34.5)
	Yes	229 (3.1)	34.9 (28.8-41.5)	57.5 (38.3-76.7)	54.4 (35.4-73.5)
**Chest pain**	No	6959 (94.6)	22.9 (21.9-23.9)	33.2 (30.1-36.4)	30.9 (28.2-33.7)
	Yes	396 (5.4)	37.9 (33.1-42.9)	52.7 (37.0-68.4)	49.6 (34.4-64.8)

Seroprevalence data are % (95% confidence interval). †Adjusted for test performance I (sensitivity 66·9% and specificity 98.2%). ‡Adjusted for test performance II as reported by manufacturer (sensitivity 71.8% and specificity 98.2%).

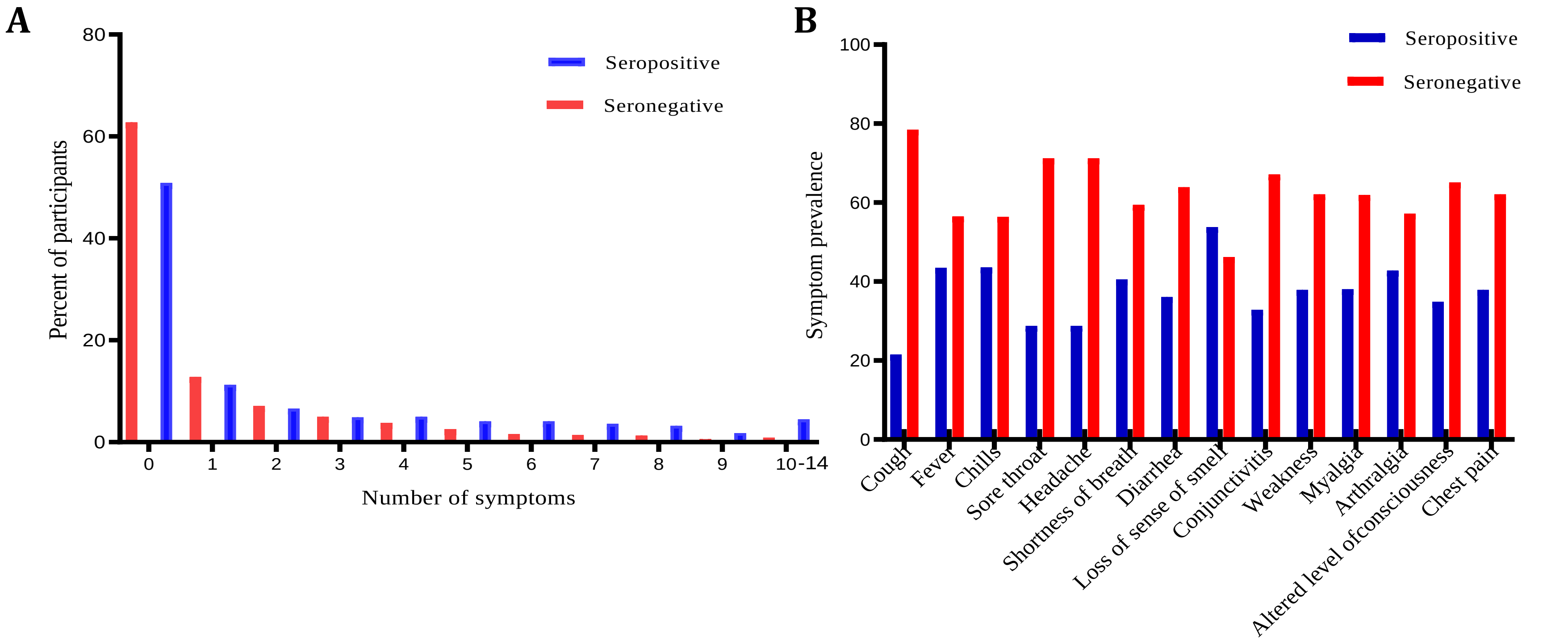

Figure S3. Proportion of participants stratified by serotypes and number of symptoms (A) and type of symptoms (B).

Table S4. Frequency of symptoms among antibody positive compared to antibody negative individuals.

Frequency (%)	Status	Total, n (%) N=7355	Antibody positive, n (%) N=1746	Antibody negative, n (%) N=5609
**Cough**	Yes	1054 (14.3)	392 (22.5)	662 (11.8)
**Fever**	Yes	786 (10.7)	341 (19.5)	445 (7.9)
**Chills**	Yes	669 (9.1)	292 (16.7)	377 (6.7)
**Loss of sense of smell**	Yes	586 (8.0)	315 (18.0)	271 (4.8)
**Sore throat**	Yes	1179 (16.0)	340 (19.5)	839 (15.0)
**Headache**	Yes	1498 (20.4)	431 (24.7)	1067 (19.0)
**Shortness of breath**	Yes	500 (6.8)	203 (11.6)	297 (5.3)
**Diarrhea**	Yes	521 (7.1)	188 (10.8)	333 (5.9)
**Conjunctivitis**	Yes	304 (4.1)	100 (5.7)	204 (3.6)
**Weakness**	Yes	1099 (14.9)	417 (23.9)	682 (12.2)
**Myalgia**	Yes	1012 (13.8)	386 (22.1)	626 (11.2)
**Arthralgia**	Yes	533 (7.2)	228 (13.1)	305 (5.4)
**Altered level of consciousness**	Yes	229 (3.1)	80 (4.6)	149 (2.7)
**Chest pain**	Yes	396 (5.4)	150 (8.6)	246 (4.4)
**Prevalence of symptoms**	Asymptomatic (0)	4409 (60.0)	888 (50.9)	3521 (62.8)
	Paucisymptomatic (1-3)	1797 (24.4)	398 (22.8)	1399 (24.9)
	Symptomatic (≥4)	1149 (15.6)	460 (26.3)	689 (12.3)

Data are n, n (%), or n/N (%)

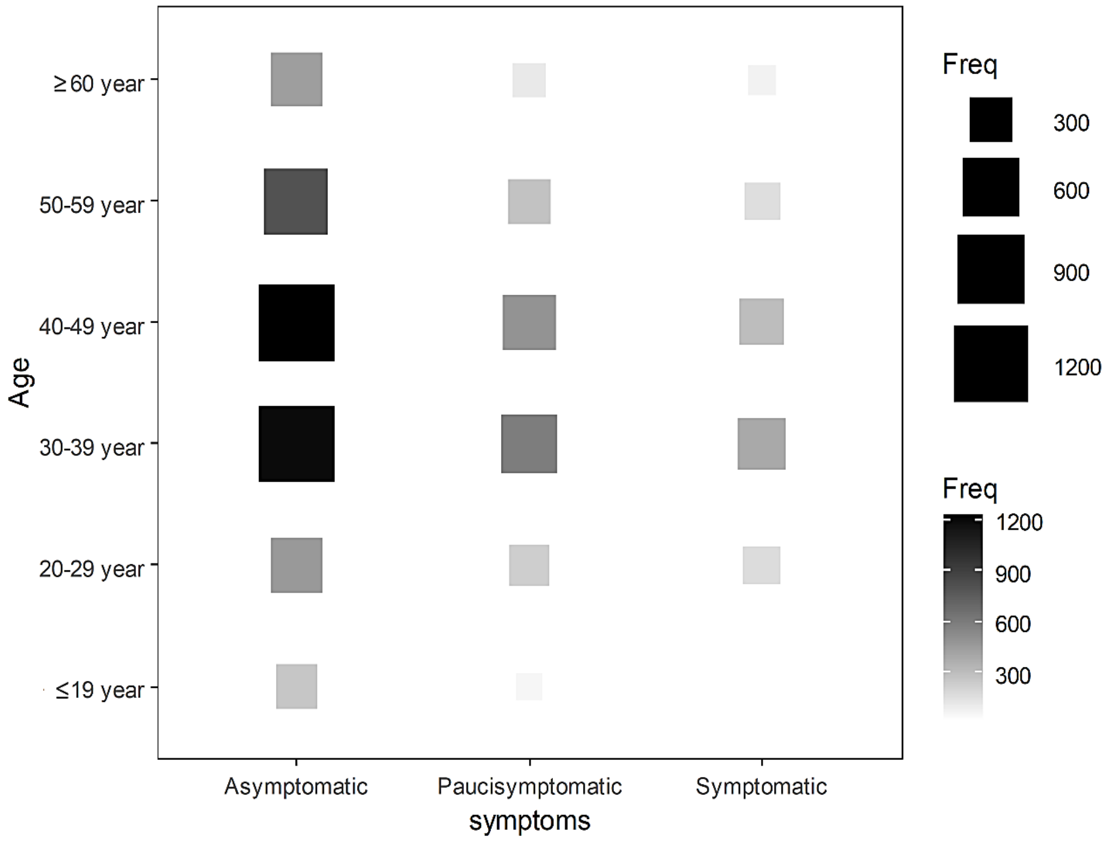

Figure S4. Distribution of symptom categories by participants' age.
